# The wearable defibrillator: a most affordable vest

**DOI:** 10.1007/s12471-017-0980-5

**Published:** 2017-04-13

**Authors:** J. R. de Groot

**Affiliations:** Heart Center, Department of Cardiology, Academic Medical Center, University of Amsterdam, Amsterdam, The Netherlands

The implantable cardioverter-defibrillator (ICD) has become a cornerstone in the prevention of sudden cardiac death in patients with ischaemic and non-ischaemic cardiomyopathy, as well as in survivors of idiopathic ventricular arrhythmias and carriers of a genetic arrhythmia syndrome. Current guidelines recommend ICD implantation in patients with a left ventricular ejection fraction ≤35%, despite optimal medical treatment for 3 months, and at least 40 days after myocardial infarction or revascularisation [1].

These delays have been implemented because ejection fraction may improve over time upon treatment, and ICD implantation shortly after myocardial infarction has been demonstrated futile, mainly due to death as a consequence of pump failure. As physicians, we can accept this. However, the fact that our patients nonetheless have a high risk of preventable *arrhythmic death* in the meantime, is harder to accept. The same risk applies to patients in whom an infected ICD system has been extracted, who face long-term antibiotic treatment during which no new device can be implanted. That is why several strategies are employed to overcome the issue of unprotected arrhythmia risk, among which monitored hospitalisation and the temporary use of a wearable cardioverter-defibrillator (WCD). In a European Heart Rhythm Association (EHRA) survey of WCD use in European countries, Lenarczyk et al. describe that less than 50% of the centres responding used the WCD [2]. The most important barriers were reimbursement issues, followed by patient compliance, and only 55% of centres reported a patient compliance of >90% per day.

In this issue of the *Netherlands Heart Journal*, Quast and colleagues report on the experience with a wearable defibrillator in two high-volume Dutch centres [3]. The report follows the initial description of the first experience with this technology in the Netherlands [4]. The majority of the patients Quast et al. report on are patients with newly diagnosed cardiomyopathy without a sufficiently long duration of optimal medical therapy, whereas in the remainder the WCD was used as a bridge to implantation in patients with a contraindication for immediate implantation due to infection or planned radiotherapy. During a median wearing time of 79 days, compliance was excellent with 23.3 h/day. Two patients received an appropriate shock, and one an inappropriate shock (all with ischaemic cardiomyopathy). This translated into an annual appropriate and inappropriate shock rate of 13.6 and 6.7%, respectively. There were no unsuccessful treatment episodes.

Can the description of a relatively low number of patients, with an established therapeutic intervention, contribute to our understanding? And what do the real-life data from the Netherlands tell us? In an era of limited health-care system funds on the one hand, and indications for ICD implantation, particularly driven by low ejection fractions (irrespective of the cause), on the other, the data that Quast and colleagues present teach us two important lessons. First, the patients concerned were indeed at a very high risk of sudden arrhythmic death. Not only was the reported annual rate of appropriate therapy for ventricular fibrillation considerably higher than in most contemporary studies, but also one of the three patients who refused ICD implantation after WCD treatment subsequently died from sudden cardiac death during follow-up (median follow-up was 1.6 years). These numbers justify any form of protection against arrhythmic death in the patient population described, and based on the data provided, sending these patients home unprotected may even be considered unethical. The direct implication of the former is that these patients, particularly those after device extraction, may indeed require long-term hospitalisation, should an alternative such as a WCD not be available. Second, of the five patients who did not refuse ICD therapy, but in whom an ICD was not implanted following WCD treatment, one received a ventricular assist device and 4 died from non-arrhythmic causes before the ICD could be implanted. This observation indicates that a considerable fraction of patients, retrospectively, had life expectancy of less than one year, which is the lower limit for ICD eligibility. Conversely, and perhaps more importantly, and in addition to the former point, a large proportion of patients (52.1%) with newly diagnosed cardiomyopathy demonstrated improvement of cardiac function, whereby the initial indication for ICD implantation was no longer present. Hence, the percentage of patients in whom the indication for ICD implantation ceased during the WCD treatment, or just by waiting, is considerable and cannot be neglected.

Similar obstacles and restrictions for the larger scale implementation of the WCD, as described by Lenarczyk et al., are likely to apply in the Netherlands as well [2]. Considering that the 79 patients that Quast et al. describe were *consecutive* patients in two high-volume centres over a period of 7 years, one can only conclude that the uptake of this wearable therapy is low. In comparison, a total of 4994 ICDs (single chamber, dual chamber and cardiac resynchronization therapy defibrillator) were implanted in the Netherlands in 2014 (EHRA Whitebook 2016).

On the use of this technology while awaiting the ejection fraction to improve, with the indication to refrain from permanent ICD implantation, hard data are lacking. It is not known how many WCDs were used countrywide over this period. Similarly, we lack data on the number of patients in whom permanent ICD implantation was deferred or not performed at all, who do not appear in the implantation statistics. What we do know, however, is that in a retrospective analysis of 1160 patients with transvenous or subcutaneous ICDs, the appropriate shock rate was considerably lower than in the patients Quast et al. describe [5]. This does not imply that the indication criteria for ICD implantation were less strict in those patients, nor can it be concluded that all patients awaiting ICD implantation are at similar risk of having arrhythmias as the patients described by Quast et al. Postponing ICD implantation beyond the guideline-directed time periods, however, clearly prevents unnecessary implantations and with that, future device-related complications. It seems safe to conclude, therefore, that the WCD is an effective and affordable bridge to destination therapy.
